# Loratadine dysregulates cell cycle progression and enhances the effect of radiation in human tumor cell lines

**DOI:** 10.1186/1748-717X-5-8

**Published:** 2010-02-03

**Authors:** Benjamin P Soule, Nicole L Simone, William G DeGraff, Rajani Choudhuri, John A Cook, James B Mitchell

**Affiliations:** 1 Radiation Biology Branch, National Cancer Institute, National Institutes of Health, 10 Center Drive, Building 10, Room B3B69, Bethesda, MD 20892, USA

## Abstract

**Background:**

The histamine receptor-1 (H1)-antagonist, loratadine has been shown to inhibit growth of human colon cancer xenografts in part due to cell cycle arrest in G2/M. Since this is a radiation sensitive phase of the cell cycle, we sought to determine if loratadine modifies radiosensitivity in several human tumor cell lines with emphasis on human colon carcinoma (HT29).

**Methods:**

Cells were treated with several doses of loratadine at several time points before and after exposure to radiation. Radiation dose modifying factors (DMF) were determined using full radiation dose response survival curves. Cell cycle phase was determined by flow cytometry and the expression of the cell cycle-associated proteins Chk1, pChk1^ser345^, and Cyclin B was analyzed by western blot.

**Results:**

Loratadine pre-treatment of exponentially growing cells (75 μM, 24 hours) increased radiation-induced cytotoxicity yielding a radiation DMF of 1.95. However, treatment of plateau phase cells also yielded a DMF of 1.3 suggesting that mechanisms other than cell cycle arrest also contribute to loratadine-mediated radiation modification. Like irradiation, loratadine initially induced G2/M arrest and activation of the cell-cycle associated protein Chk1 to pChk1^ser345^, however a subsequent decrease in expression of total Chk1 and Cyclin B correlated with abrogation of the G2/M checkpoint. Analysis of DNA repair enzyme expression and DNA fragmentation revealed a distinct pattern of DNA damage in loratadine-treated cells in addition to enhanced radiation-induced damage. Taken together, these data suggest that the observed effects of loratadine are multifactorial in that loratadine 1) directly damages DNA, 2) activates Chk1 thereby promoting G2/M arrest making cells more susceptible to radiation-induced DNA damage and, 3) downregulates total Chk1 and Cyclin B abrogating the radiation-induced G2/M checkpoint and allowing cells to re-enter the cell cycle despite the persistence of damaged DNA.

**Conclusions:**

Given this unique possible mechanism of action, loratadine has potential as a chemotherapeutic agent and as a modifier of radiation responsiveness in the treatment of cancer and, as such, may warrant further clinical evaluation.

## Background

It is well established that the effects of radiation varies as a function of cell cycle position [1]. Specifically, cells in G2/M phase are particularly susceptible to the effects of radiation. Because of this, agents that alter cell cycle progression are often potent radiation modifiers [2]. Normal cell cycle regulation is mediated by several proteins that are responsive to both intra- and extracellular stimuli. It has been demonstrated that the commonly used antihistamine loratadine (ethyl4-(8-chloro-5,6-dihydro-11H-benzo[5,6]cyclohepta [1,2-b]pyridin-11-ylidene)-1-piperidinecarboxylate), an antagonist of histamine receptor-1, induces a cell cycle arrest in G2/M by interfering with the activity of these regulatory proteins [3]. Although a comprehensive mechanism was not elucidated, in these prior studies loratadine treatment resulted in anti-tumor effects.

Progression through the cell cycle is regulated by a complex network of proteins that monitor the health of the cell. This mechanism serves to protect cells from potentially lethal stressors by temporarily halting cell cycle progression to allow time for repair of damaged cell components, especially damage involving DNA. For example, it is well known that DNA damage induced by radiation results in cell cycle block in G2/M during which time the DNA repair machinery attempts to correct the damage. If the damage is repaired, cells are released from the cell cycle block and are allowed to divide. Persistent DNA damage may result in cell death initiated by other surveillance mechanisms. In eukaryotic cells, the G2/M checkpoint is controlled by several proteins including cell division cycle 2 (Cdc2) and Cyclin B [4]. Cdc2 is inactivated by phosphorylation (Tyr-15, Thr-14) and activated by Cdc25C-mediated dephosphorylation [5]. Cdc25C, in turn, is regulated by 14-3-3, which inhibits nuclear translocation of Cdc25C, and Chk1 phosphorylation, which allows 14-3-3 binding to occur [6]. Chk1 inhibition has been associated with increased cytotoxicity of DNA damaging drugs [7-12], and in our lab with increased sensitivity to the effects of radiation (unpublished data). Recently, loratadine has also been shown to cause Cdc2-associated G2/M arrest by interfering with Chk1 and Cdc25C signaling [3]. It is likely that the anti-tumor effects of loratadine observed in other studies result, at least in part, from this activity.

Since G2/M is a particularly radiosensitive phase of the cell cycle, it is logical to suggest that the induction of a cell cycle block in G2/M by loratadine would enhance radiation-induced cytotoxicity, however this has not yet been studied. This study was initiated to determine whether loratadine modifies the effect of radiation on cell survival and, if so, to elucidate the mechanism underlying that effect.

## Methods

### Cell Culture Studies

**Figure 1:**
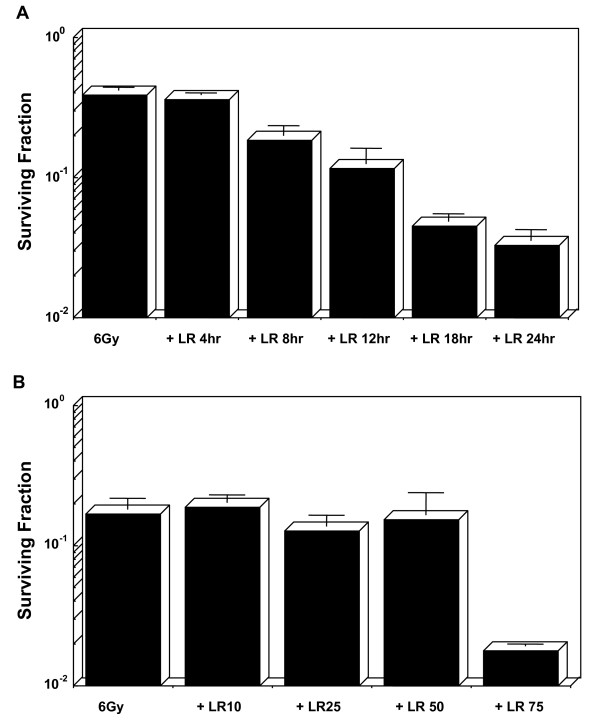
**Effect of Loratadine Dose and Exposure Time on Response to Radiation**. (A) Cells were treated with 75 μM loratadine for various times prior to irradiation to 6 Gy. The radiation modifying effect increased with exposure time. Toxicity of loratadine alone was minimal until exposure exceeded 18 hrs. (B) Cells were treated with loratadine (0, 10, 25, 50, 75, 150, 300, and 450 μM) for 24 hrs prior to irradiation. A radiation modifying effect was only observed with a 75 μM dose. Toxicity of loratadine alone increased with dose and 100% of the cells were killed at doses above 75 μM (data not shown). Cell survival is corrected for the toxicity of loratadine alone. The figure represents the mean ± SD for 3 experiments.

HT29 (human colon carcinoma) and DU145 (human prostate carcinoma) were purchased from American Type Culture Collection (Manassas, VA). SF295 (human glioblastoma) were a gift from Dr. Kevin Camphausen. SF295 cells were grown in DMEM, and all other cell lines were grown in RPMI 1640. All media contained 10% heat-inactivated fetal bovine serum and antibiotics. For cell survival studies, cells were plated (5 × 10^5 ^cells/100 mm plastic petri dish) and incubated for 16 hours at 37°C. Loratadine was dissolved in 0.1% DMSO then added at various concentrations to the exponentially growing cells in complete medium and the cells were incubated at 37°C for 24 hour. DMSO (0.1%) was also added to control cells. Most studies used a loratadine concentration of 75 μM [3]; the only exception was studies shown in Figure 1B where a range of loratadine concentrations were used (10-450 μM). Some studies involved the use of plateau phase cultures. For these studies, cells were allowed to grow to confluence and maintained in confluence without medium change for 3 days after which they were treated with loratadine (75 μM) as described above. Flow cytometery studies confirmed that these cultures were enriched in cells in G1 phase. Following incubation cells with or without loratadine, cells were treated with varying doses of radiation using an Eldorado 8 cobalt-60 teletherapy unit (Theratronics International Ltd. Kanata, Ontario, Canada) at dose rates of 2.0-2.5 Gy/min. Control radiation survival curves were conducted in parallel. Immediately after irradiation, cells were trypsinized, counted, plated, and incubated for 10-14 days for macroscopic colony formation. Colonies were then fixed with methanol/acetic acid (3:1) and stained with crystal violet. Colonies with >50 cells were scored and cell survival determined after correcting for the plating efficiency and for loratadine cytotoxicity alone. For radiation studies, a dose modification factor (DMF) was determined by taking the ratio of radiation doses at the 10% survival level (control radiation dose divided by the drug treated radiation dose). DMF values > 1 indicate enhancement of radiosensitivity. Some studies involved cisplatin exposure to cells for 1 hour with or without a 24 hour pre-treatment with loratadine. Following treatment, the cells were processed for colony formation as described above.

### Immunoblot Analysis for γH2AX

Cells were lysed in 10 mM HEPES pH 7.9, 1.5 mM MgCl2, 10 mM KCl with 0.5 mM DTT and 1.5 mM PMSF with complete protease inhibitor cocktail (Roche Applied Science, Indianapolis, IN). Histones from the nuclear pellet were extracted in 0.2 mol/L sulfuric acid by incubating samples on ice for 4-6 hours. After centrifugation, acid-soluble histones were transferred to fresh tubes and 9 volumes of ice cold acetone were added. Histones were precipitated at -20°C overnight and were pelleted by centrifugation at 14,000 rpm for 10 min at 4°C. Supernatant was discarded and pellets were air-dried. Histones were solubilized in 4 mol/L urea and protein concentration was determined by BioRad DC protein assay. Histones were separated on 18% Tris-Glycine gels (Invitrogen, Carlsbad, CA) by loading 20 μg samples and transferred to nitrocellulose membrane using iBlot Dry Blotting System from Invitrogen (Carlsbad, CA). Membranes were incubated overnight at 4°C with mouse monoclonal anti-phospho Histone H2AX (Ser139), clone JBW301 (1:10,000) from Millipore (Billerica, MA), washed 3 times with PBS-T and incubated with HRP-conjugated anti-mouse antibody from Santa Cruz Biotechnology, Inc. (Santa Cruz, CA). γH2AX was visualized by ECL detection kit (Perkin Elmer, Waltham, MA) using Fluor Chem SP imager (Alpha Innotech, San Leandro, CA). Membranes were stripped using Re-Blot Plus mild antibody stripping solution (Millipore; Billerica, MA) and reprobed with 1:1000 rabbit antiserum to histone H2A (acidic patch) from Millipore (Billerica, MA) to ascertain uniform loading. Signal intensities were normalized to their loading control H2A and expressed as fold change compared to controls.

### Pulsed-Field Gel Electrophoresis

DNA was prepared for electrophoresis by the methods of Schwartz and Cantor [13] and Gardiner et al. [14] as modified by Ager and Dewey [15] and Stamato and Denko [16]. After loratidine treatment (or x-irradiation for a positive control), the cells were trypsinized, rinsed in cold PBS, and resuspended in PBS at 10^7 ^per ml. An equal volume of 1% low gelling temperature agarose was added, and the cell suspension was drawn into ^3^/_32 _inch (i.d.) silicone tubing with a syringe. Both ends of the tubing were clamped, and the tubing was immersed in an ice bath to rapidly solidify the agarose. The agarose was then extruded from the tubing, cut into 5 mm lengths, and these "plugs" were placed into 1.5 ml centrifuge tubes. This procedure results in approximately 10^5 ^cells per 5 mm plug. DNA was purified by incubating at 55°C in ESP buffer (0.5 M EDTA, 1% Sarkosyl, and 50 μg/ml proteinase K) for 24 hr. The plugs were then rinsed in TE buffer (10 mM Tris, 1 mM EDTA) for 24 hr with three buffer changes. RNA was digested by incubation with 0.1 μg/ml boiled RNAse A in TE buffer for 2 hr at 37°C.

0.8% agarose gels were cast in 0.5× TBE (1× TBE = 90 mM Tris, 90 mM boric acid, 2.5 mM EDTA with 0.5 μg/ml ethidium bromide). Agarose plugs were loaded into 2 × 6 × 5 mm wells, and the wells were sealed with melted agarose. Electrophoresis was carried out for 24 hr at 56 volts (4 volts/cm), with a 3:1 ratio of forward to reverse pulse time. The initial forward pulse time was 7.5 seconds (reverse pulse 2.5 seconds), increasing to a final forward pulse time of 90 seconds (final reverse pulse 30 seconds). The running buffer (0.5× TBE) was re-circulated and cooled to maintain a temperature of 12-15°C. These electrophoresis conditions were chosen based on methods of Stamato and Denko [16], and the desire to keep the released DNA concentrated in a narrow band to facilitate quantification.

Quantification was done by densitometry using a FluorChem gel documentation system (Alpha Innotech, San Leandro, CA) and AlphaEaseFC software (Alpha Innotech, San Leandro, CA). Each band in the gel was outlined manually and the density determined. The results are expressed as "%DNA released," determined by dividing the density of the released DNA band by the density of the total DNA in the lane (the released DNA band plus the unreleased DNA remaining in the well).

### Cell Cycle Analysis

The effect of loratadine on cell cycle distribution was analyzed by flow cytometry by propidium iodide staining after treating cells with the drug for 24 hour. Briefly, cells were trypsinized, washed with PBS and fixed in 70% ethanol overnight. Cells were pelleted and nuclei were isolated by pepsin/HCl digestion followed by treatment with 10 mmol/L borate (pH 8.6) to neutralize the acid. Cells were then incubated with FITC-labeled anti-human IgG and PI staining. Cell cycle data were collected on BD FACSCalibur Flow Cytometer (San Jose, CA) and analyzed using CellQuest/MOD-Fit software (Verity Software House, Topsham, ME).

### Western Blot Analysis

The cells were lysed in RIPA buffer (Santa Cruz Biotechnology, Inc. Santa Cruz, CA) containing protease inhibitor cocktail and phosphatase inhibitors (Roche Applied Science, Indianapolis, IN). The samples were incubated in the lysis buffer on ice for 30 minutes, centrifuged at 14000 rpm in a refrigerated centrifuge for 30 minutes and the supernatant collected. The samples were kept at 40°C if used on the same day or frozen at -70°C for storage. Protein concentration was determined with Dc Protein Asssay kit (Bio-Rad, Hercules, CA). 40 μg of protein was separated on 4-20% Tris-Glycine gels (Invitrogen, Carlsbad, CA) and transferred to nitrocellulose membrane using iBlot Dry Blotting System from Invitrogen (Carlsbad, CA). Non specific protein binding was blocked by incubating the membranes for 1 hour in 3% blocking grade non fat dry milk (Bio-Rad, Hercules, CA) in TBST. The membranes were then left overnight at 40°C in the primary antibody at a dilution of 1:1000 for rabbit monoclonal anti pChk1 (Ser 345) (Cell Signaling Technology, Inc., Danvers, MA), 1:200 for mouse monoclonal anti Chk1 (Santa Cruz Biotechnology, Inc. CA), 1:1000 for mouse monoclonal anti Cyclin B (BD Biosciences, Bedford MA); and 1:5000 for mouse monoclonal anti Actin (Millipore, Billerica, MA). The membranes were washed thrice in TBST and incubated for 1 hour in horseradish peroxidase conjugated secondary antibody (Santa Cruz Biotechnology, Inc. Santa Cruz, CA) at a dilution of 1:2000. The proteins were then visualized by chemiluminescence (Western Lightning Chemilumiscence Reagent Plus, Perkin Elmer, Waltham, MA or ECL Advance Western Blotting Detection Kit, GE Lifesciences, Pittsburg, PA) using Fluor Chem SP imager (Alpha Innotech, San Leandro, CA). Fold change in protein expression was expressed as a ratio calculated by dividing the specific protein band density with the actin band density (loading control), and then normalized to the control.

### Statistics

All experiments were performed a minimum of three times. In some cases, the plots represent the average of these experiments. For some experiments, representative results are represented. Whether the plot represents the average of several experiments or a representative experiment has been indicated for each figure. When present, error bars represent the standard error of the mean. Dose modifying factors (DMF) were calculated for clonogenic survival assays.

## Results

### Loratadine Dose Response and Time Course in Radiation-treated Cells

HT29 cells treated with loratadine (75 μM) 4, 8, 12, 18, and 24 hours prior to irradiation (6 Gy) demonstrated that the radiation modifying effect of loratadine increased with increasing exposure time prior to irradiation (Figure 1A). The toxicity of loratadine alone was minimal until exposure time exceeded 18 hours. For all experiments, cell survival was assessed using a standard clonogenic assay corrected for the toxicity of loratadine alone. HT29 cells were then treated with loratadine (0, 10, 25, 50, 75, 150, 300, and 450 μM) for 24 hours prior to irradiation (6 Gy). Loratadine decreased cell survival by one log after administration of a 75 μM dose but no effect was observed at lower doses (Figure 1B). The cytotoxicity of loratadine alone increased with increasing dose and was noted to increase markedly at the 75 μM dose as well. Doses of loratadine higher than 75 μM killed 100% of the cells (data not shown).

### Effect of Loratadine on Radiation Dose Response

**Figure 2:**
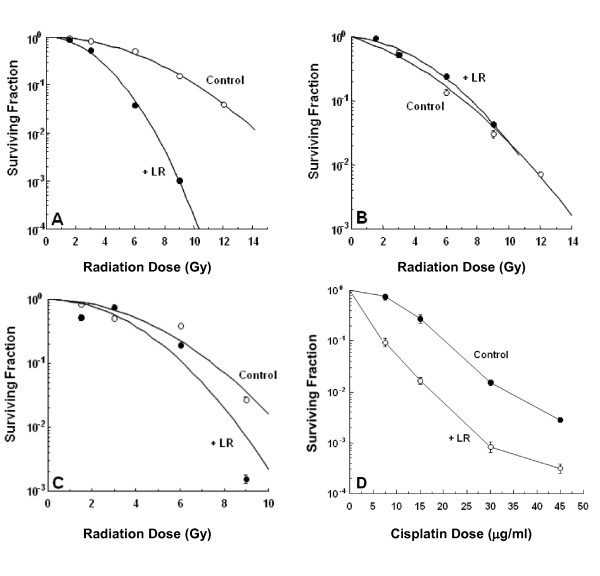
**Effect of Loratadine on Radiation or Cisplatin Dose Response**. HT29 cells in log phase growth were treated with loratadine (75 μM) for 24 hrs prior to irradiation (A) or for 24 hrs after irradiation (B) to 0, 1.5, 3, 6 or 9 Gy or 12 Gy (controls only). A radiation DMF of 1.95 ± 0.07 was observed in cells pre-treated with loratadine. There was no significant radiation modification by loratadine treatment after irradiation. (C) HT29 cells in plateau phase growth pre-treated with loratadine (75 μM, 24 hrs) demonstrated a radiation DMF of 1.3 ± 0.16. Solid circles = loratadine + radiation, open circles = radiation alone. (D) HT29 cells in log phase growth were pre-treated with loratadine (75 μM, 24 hrs) prior to treatment with Cisplatin (7.5, 15, 30, or 45 μg/ml for 1 hr). A DMF of 2.6 ± 0.14 was observed. Open circles = loratadine + cisplatin, solid circles = cisplatin alone. Cell survival was assessed by clonogenic assay and corrected for the toxicity of loratadine alone. The figure represents the mean ± SD for 3 experiments.

HT29 cells in log phase growth were treated with loratadine (75 μM) for 24 hours prior to irradiation (0, 1.5, 3, 6, or 9 Gy, or 12 Gy for controls only). A radiation dose response was clearly demonstrated with enhancement of the radiation-induced cytotoxicity by loratadine at all radiation doses (Figure 2A) resulting in a radiation dose modification factor (DMF) of 1.95 ± 0.07 compared to cells not treated with loratadine. In contrast, HT29 cells in log phase growth treated with loratadine (75 μM) for 24 hours after irradiation (0, 1.5, 3, 6, 9, or 12 Gy) appeared to be minimally protected from radiation-induced cytotoxicity (Figure 2B). HT29 cells were allowed to reach plateau phase in culture and were then treated with loratadine (75 μM) for 24 hours prior to irradiation (0, 1.5, 3, 6, or 9 Gy). This resulted in a radiation DMF of 1.3 ± 0.16 compared to cells not treated with loratadine (Figure 2C).

### Radiation Dose-modifying Effect of Loratadine in Other Cell Types

HT29, SF295, and DU145 cells in log phase growth were treated with loratadine (75 μM) for 24 hours prior to irradiation (6 Gy) and cell survival was assessed using a clonogenic assay. Loratadine alone was more toxic to SF295 cells (17% survival) than HT29 cells (68% survival) but enhanced the radiation response in both cell lines (data not shown). Despite significant toxicity to DU145 cells of loratadine alone (45% survival), no increase in susceptibility to radiation-induced cytotoxicity was seen in loratadine-treated cells.

### Effect of Loratadine on Cisplatin-treated Cells

HT29 cells treated with loratadine (75 μM) for 24 hours prior to treatment with Cisplatin (7.5, 15, 30, or 45 μg/ml for 1 hour). A Cisplatin dose response was clearly demonstrated with enhancement of the cisplatin-induced cytotoxicity by loratadine at all radiation doses (Figure 2D) resulting in a DMF of 2.6 ± 0.14 for loratadine.

### Effect of Histamine on Radiation Modification by Loratadine

To establish whether the observed effects of loratadine were being mediated by antagonism of the H1-receptor, HT29 cells were treated with loratadine with and without exogenous histamine. Exposure to histamine (100 or 1000 μM) alone for 15 minutes did not alter survival (data not shown). At both doses a cell survival of 99% was observed by clonogenic assay. Likewise, histamine did not modify the response to radiation as there was no significant difference between cells exposed to 9 Gy alone compared to those that were pretreated with histamine.

### Effect of Loratadine and Radiation on DNA-Repair Proteins

**Figure 3:**
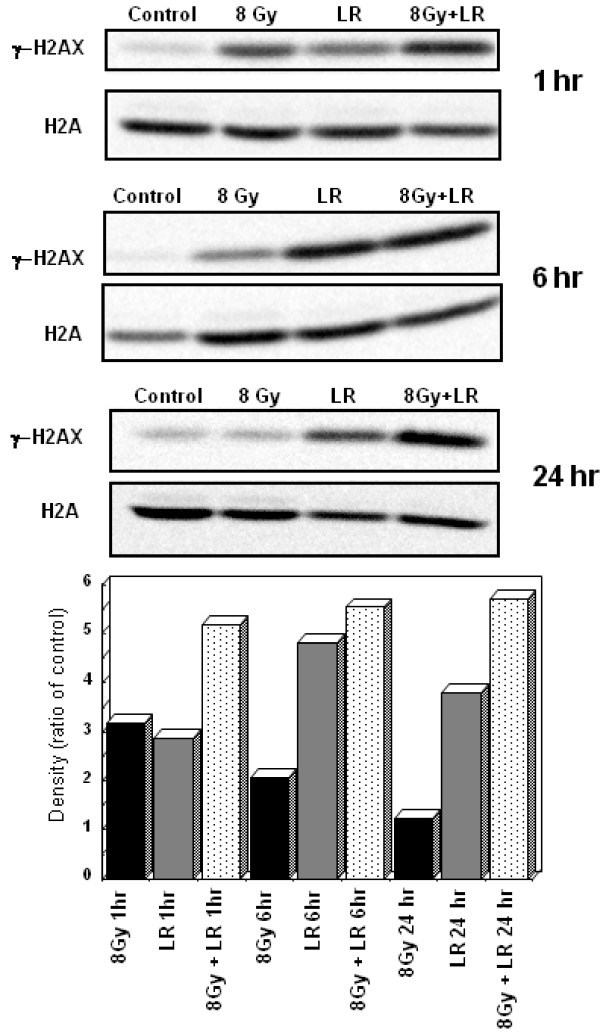
**Effect of Loratadine and Radiation on DNA Repair Proteins**. HT29 cells were either treated with loratadine (75 μM, 24 hrs) prior to exposure to 8 Gy radiation, or treated with loratadine or radiation alone. γH2AX expression, determined by western blot at 1, 6, and 24 hrs after irradiation, increased within 1 hr after irradiation and returned to baseline by 24 hrs. Loratadine treatment enhanced this expression at 1 hr and resulted in persistent expression at 24 hrs. Loratadine alone also increased γH2AX expression with maximal expression at 6 hrs. The graph represents the ratio of the densitometric value of the sample compared to control for a single representative experiment, LR = loratadine-treated.

**Figure 4:**
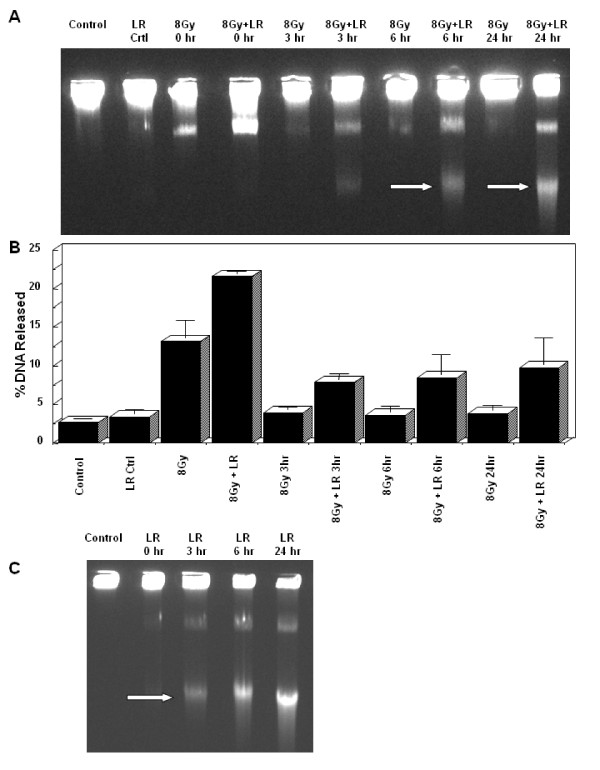
**Effect of Loratadine and Radiation on DNA Damage**. HT29 cells were either treated with loratadine (75 μM, 24 hrs) prior to exposure to 8 Gy radiation, or treated with loratadine or radiation alone. Cells were analyzed by pulsed-field gel electrophoresis at 0, 3, 6, and 24 hrs. (A) Radiation-induced DNA fragments were evident immediately following irradiation and resolved by 24 hrs. Loratadine-treated irradiated cells demonstrated increased and persistent DNA fragmentation and an additional band corresponding to smaller DNA fragments (arrows). (B) Densitometry analysis confirmed increased DNA fragments in irradiated and loratadine-treated cells. (C) Loratadine alone induced DNA fragmentation and an additional band corresponding to smaller DNA fragments (arrow). The graph represents the densitometric value of the sample for a single representative experiment, LR = loratadine-treated.

γH2AX recruitment was measured by western blot in HT29 cells treated with loratadine (75 μM) for 24 hours prior to irradiation (6 Gy) then collected at 1, 6 and 24 hours after irradiation. One hour post-irradiation, γH2AX was increased in radiation-treated samples compared to unirradiated control (Figure 3). Commensurate with DNA repair, this signal decreased over time and returned to baseline by 24 hours post-irradiation. At one hour post-irradiation, the loratadine-treated irradiated sample demonstrated more γH2AX signal than the radiation-only sample. In contrast to the radiation-only cells, the γH2AX signal in the cells treated with loratadine and radiation remained elevated at 6 and 24 hours without evidence of diminution. Cells treated with loratadine alone also demonstrated increased γH2AX signal which increased at 6 hours but diminished by 24 hours after treatment. Using the same experimental design, HT29 cells were analyzed by pulsed-field gel electrophoresis at 0, 3, 6, and 24 hours post-irradiation. As shown in Figure 4A, radiation-induced DNA fragments (8 Gy) were evident within 1 hour following irradiation and resolved by 24 hours. Loratadine-treated irradiated cells (LR+8 Gy) demonstrated increased DNA fragmentation compared to radiation alone, and this increase persisted through 24 hours. An additional band corresponding to smaller DNA fragments (arrows) was also seen in loratadine-treated cells. Densitometry analysis confirmed increased DNA fragments in irradiated cells, and a further increase in loratadine-treated irradiated cells (Figure 4B). Loratadine alone (LR) induced DNA fragmentation (Figure 4C) and also produced the additional band corresponding to smaller DNA fragments (arrow).

### Effect of Loratadine on In Vitro Cell Cycle Progression

**Figure 5:**
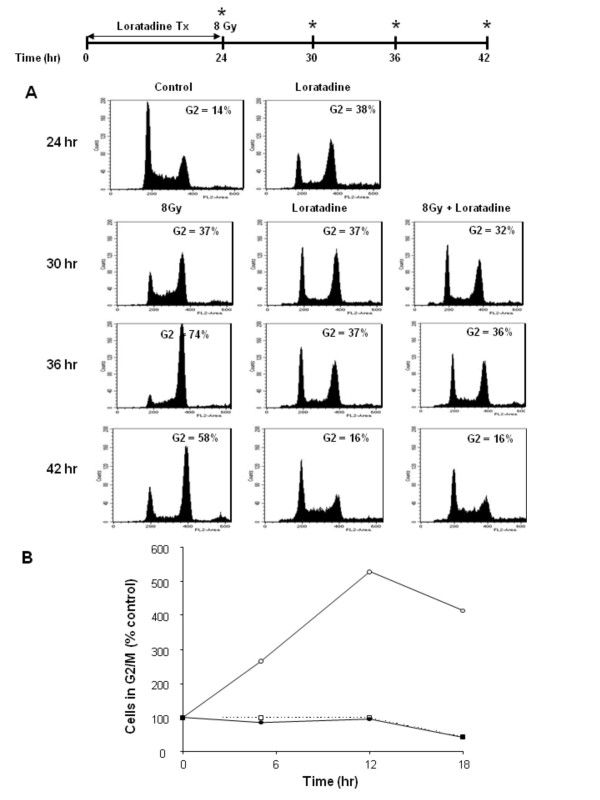
**Effect of Loratadine and Radiation on Cell Cycle Progression**. HT29 cells were either treated with loratadine (75 μM, 24 hrs) prior to exposure to 8 Gy radiation, or treated with loratadine or radiation alone. Cell cycle progression was analyzed by flow cytometry. (A) After 24 hr treatment with loratadine, cells exhibited a G2 block (38%) which persisted through 36 hrs and resolved by 42 hrs. Irradiated cells also exhibited a G2 block which peaked (74%) at 12 hrs after irradiation and began to decrease 18 hrs after irradiation. Loratadine abrogated the radiation-induced G2 block at 12 hrs post-irradiation and by 18 hrs had returned to baseline. (B) Irradiation alone (open circle) increased the percentage of cells in G2/M but did not increase the percentage of loratadine-treated cells (solid circle) in G2/M compared to loratadine treatment alone (open square). The figure represents a single representative experiment.

HT29 cells treated with loratadine (75 μM) for 24 hours. Loratadine was then washed off and cells were irradiated (8 Gy). Cell cycle progression was analyzed by flow cytometry after loratadine treatment, then again 6, 12, and 18 hours after irradiation. After 24 hour treatment with loratadine alone, cells exhibited a G2 block (from 14 to 37%) (Figure 5A). This G2 block persisted for 12 hours (hour 36) and returned to baseline by 18 hours after treatment (hour 42). Six hours after irradiation alone (hour 30) cells also exhibited a G2 block (8 Gy) which was similar in magnitude to loratadine-treated cells. The radiation-induced G2 block increased from 14 to 74% by 12 hours after irradiation and began to decrease 18 hours after irradiation (from 74 to 58%). Interestingly, despite inducing a G2 block, loratadine-treated cells clearly dominated the cell cycle delay and radiation with loratadine did not cause additional cell cycle delays. As shown in Figure 5B, the percent of cells in G2 did not increase following radiation in loratadine-treated cells. In contrast to cells exposed to radiation alone, the loratadine-treated irradiated cells had returned to a more normal cell cycle distribution within 18 hours of the removal of loratadine.

### Effect of Loratadine on Cell Cycle-associated Proteins

**Figure 6:**
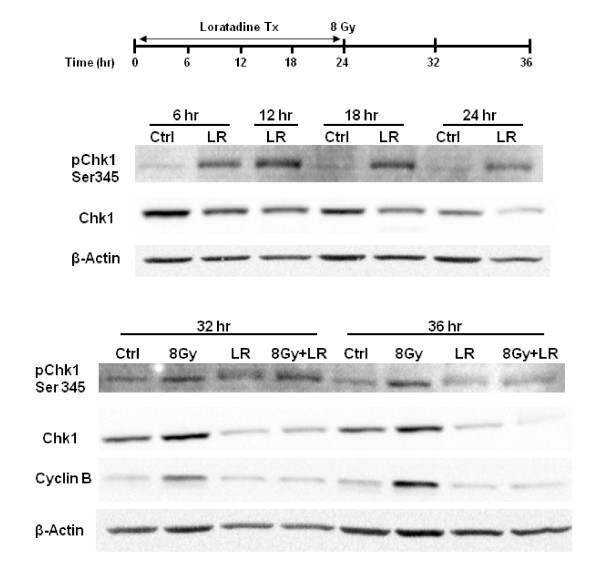
**Effect of Loratadine and Radiation on Cell Cycle Associated Proteins**. In HT29 cells, activated pChk1^ser345 ^increases in response to loratadine (LR, 75 μM) within 6 hrs after exposure, peaks at 12 hrs and returns to baseline by 36 hrs. Total Chk1 decreases after exposure and remains depressed at 36 hrs. In irradiated cells, both pChk1^ser345 ^and Chk1 are increased at 8 and 16 hrs post-irradiation (8 Gy, 32 and 36 hr). Loratadine does not alter the radiation-induced increase in pChk1^ser345 ^at 8 hrs post-irradiation (8 Gy+LR, 32 hr) but abrogates the increase seen at 16 hrs (8 Gy+LR, 36 hr). Cyclin B1 increases in irradiated cells at 8 and 16 hrs post-irradiation (8 Gy, 32 and 36 hr) but in cells treated with loratadine (LR, 8 Gy+LR) Cyclin B expression falls below that seen in controls. The figure represents a single representative experiment.

Western blots were performed to detect total Chk1, phosphorylated Chk1 (pChk1^ser345^) and Cyclin B in HT29 cells treated with loratadine (75 μM) for 24 hours prior to irradiation (8 Gy). pChk1^ser345 ^increases in response to loratadine (LR) within 6 hours after exposure, peaks at 12 hours and returns to baseline by 36 hours (Figure 6). Chk1 progressively decreases after exposure and remains depressed below baseline expression at 36 hours. In irradiated cells (8 Gy), both pChk1^ser345 ^and Chk1 are increased at 8 and 16 hours post-irradiation. Loratadine does not significantly alter the radiation-induced increase in pChk1^ser345 ^at 8 hours post-irradiation (8 Gy+LR) but in contrast to cells exposed to radiation only, pChk1^ser345 ^expression returns to control levels by 12 hours post-irradiation. Furthermore, Chk1 levels in cells exposed to radiation and loratadine are markedly decreased compared to cells exposed to radiation alone and even compared to controls. Cyclin B increases in irradiated cells at 8 and 16 hours post-irradiation but this response is abrogated in cells treated with loratadine.

## Discussion

In this study, treatment with loratadine enhanced the cytotoxic effect of radiation. This effect was both time and dose dependent and occurred optimally when cells were treated with 75 μM loratadine for 24 hours prior to irradiation. Loratadine exhibited significant cytotoxicity alone and a narrow therapeutic window with little to no effect below 75 μM and profound toxicity above that dose. This radiation-enhancing effect was observable in several cell lines including colon cancer, glioblastoma, and prostate cancer lines.

The mechanism by which radiation-enhancement occurred, however, appeared to be somewhat more complex than predicted based on previous studies. As might be expected, the action of loratadine on its putative target, the H1-receptor, did not appear to be play a mechanistic role as incubation with histamine did not prevent the loratadine-mediated radiosensitization. As has been previously shown [3], loratadine alone results in Chk1 activation leading to an increase in the percentage of cells in the G2/M phase of the cell cycle. Since the G2/M phase of the cell cycle is one of the most sensitive to radiation [17], this could explain some of the increased radiation-induced cytotoxicity observed with loratadine pre-incubation. Likewise, enrichment of the cells in G2/M phase may also explain some of the increase in susceptibility to radiation-induced DNA damage as reflected in the increase in both DNA strand breaks detected on pulsed-field gel electrophoresis and in the increased expression of the DNA repair protein γH2AX compared to cells treated with radiation alone. Our results confirm the finding of Chen et al that loratadine activates Chk1 leading to accumulation of cells in G2/M phase of the cell cycle. Our data suggest that other parts of the cell cycle are also affected since the percentage of cells in G2/M never increased beyond 38% while radiation and drugs such as cisplatin can lead to increases in G2/M of 80% or more after 12 hours of exposure [2]. Additionally, what is novel about our findings is that loratadine exposure leads to an abrogation of the G2/M checkpoint induced by radiation. Loratadine exposure appears to result in aberrant Chk1 control hence releasing them back into the cell cycle with persistent DNA damage. This may alter the ability of cells to repair additional DNA damage such as that induced by radiation contributing to the increased radiation sensitivity observed in loratadine-treated cells. One possible mechanism of this negated Chk1 response may be related to the decreased expression of total Chk1 and Cyclin B proteins after prolonged exposure to loratadine (Figure 6).

This finding is further supported by the enhancement and persistence of both the DNA fragments detected by pulsed-field gel electrophoresis and the γH2AX expression. The persistence of DNA damage may also account for the appearance of the second band of fragmented DNA that was observed on pulsed-field gel electrophoresis in loratadine treated cells (Figure 4). It is possible that these fragments represent further damage induced by ongoing attempts to repair DNA while the cell is actively progressing through the cell cycle, although this remains to be shown. It is clear, however, that DNA repair proteins, such as γH2AX, are appropriately recruited to sites of damage initially and are detected in cells treated with loratadine alone, and in loratadine treated and untreated irradiated cells (Figure 3). This recruitment is downregulated within 24 hours as DNA repair is completed in cells exposed to radiation alone. In loratadine treated cells, however, there is a persistence of this signal beyond 24 hours and well after the cells have re-entered the cell cycle. This likely results from the persistence of DNA damage as mentioned above and strongly suggests that cells are prematurely re-entering the cell cycle with persistent DNA damage that is actively undergoing attempts at repair.

Finally, loratadine also generates DNA damage on its own which induces DNA repair mechanisms in the cell such as γH2AX. The pulsed field gels demonstrate the presence of double strand breaks, however since additional lower molecular weight DNA is also present, other types of DNA damage must be occurring. This DNA damage occurs at doses of 75 μM and above and it appears that this damage is required for radiosensitization as lower concentrations did not result in an increase in DNA damage or radiation-induced cytotoxicty. Since the flow DNA histograms (Figure 5A) did not show an increased sub-G1 peak after loratadine exposure, it does not appear that an increase in apoptosis explains the increase in radiation sensitivity. Given that loratadine pre-treatment also enhanced the toxicity of cisplatin, another DNA-damaging agent, it is logical to suggest that the abrogation of the G2/M delay is a crucial mechanism underlying the loratadine-induced increase in cytotoxicty.

## Conclusions

Loratadine enhancement of the cytotoxic effect of radiation is both dose and time-dependent. The mechanism underlying this effect is multifactorial and involves an early promotion of G2/M cell cycle blockade which enhances radiation sensitivity, followed by abrogation of the radiation-induced G2/M arrest and premature release of DNA-damaged cells back into the cell cycle. Loratadine-induced DNA damage is also observed and is likely additive to the radiation-induced damage. Given this unique potential mechanism of action, loratadine is a potentially promising radiation modifying drug.

## Abbreviations

DMF: Dose Modifying Factor; Gy: Gray; H2AX: histone 2AX; γH2AX: phosphorylated histone 2AX; HT29: human colon cancer cell line; DU145: human prostate cancer cell line; SF295: human glioblastoma cell line.

## Competing interests

The authors declare that they have no competing interests.

## Authors' contributions

BPS - design and performance of lab experiments, writing and editing of manuscript.

NLS - design and performance of lab experiments, writing and editing of manuscript.

WGD - design and performance of lab experiments, editing of manuscript.

RC - performance of lab experiments.

JAC - design and performance of lab experiments, editing of manuscript.

JBM - design and performance of lab experiments, writing and editing of manuscript.

All authors have read and approved the final manuscript.
